# Risk calculator for predicting postoperative pneumonia after gastroenterological surgery based on a national Japanese database

**DOI:** 10.1002/ags3.12248

**Published:** 2019-04-22

**Authors:** Yoshio Takesue, Hiroaki Miyata, Mitsukazu Gotoh, Go Wakabayashi, Hiroyuki Konno, Masaki Mori, Hiraku Kumamaru, Takashi Ueda, Kazuhiko Nakajima, Motoi Uchino, Yasuyuki Seto

**Affiliations:** ^1^ The Japanese Society of Gastroenterological Surgery Tokyo Japan; ^2^ National Clinical Database Tokyo Japan

**Keywords:** gastroenterological surgery, National Clinical Database, pneumonia, postoperative complication, risk model

## Abstract

**Background:**

The aim of the present study was to develop a risk calculator predictive of postoperative pneumonia in patients undergoing gastroenterological surgery.

**Methods:**

We analyzed data from 382 124 patients undergoing eight main gastroenterological surgeries between 2011 and 2013 using the National Clinical Database in Japan. A risk model was developed using multivariate logistic regression analysis with patient data from 2011 to 2012 (n = 247 604) and validated using data from 2013 (n = 134 520).

**Results:**

Pneumonia was observed in 11 105 patients (2.9%). After the input of significant primary disease and surgical procedures, 18 patient characteristics including gender, chronic obstructive pulmonary disease, sepsis, and need for any assistance in the activities of daily living, six laboratory parameters, and two intraoperative factors were used for risk calculation. Area under the receiver‐operating characteristic curve was 0.822 (95% confidence interval, 0.817‐0.826) in the derivation group and 0.826 (0.819‐0.832) in the validation group.

**Conclusion:**

The risk calculator accurately predicted the occurrence of pneumonia following gastroenterological surgery.

## 1 INTRODUCTION

Pneumonia is the third leading cause of postoperative infectious complications, following surgical site infections (SSI) and urinary tract infections.^1^ Incidence of postoperative pneumonia after cardiac surgery has been reported to be between 2.1% and 3.3%.^2^, ^3^, ^4^ This is higher than in non‐cardiac surgery, which has a postoperative pneumonia incidence of between 0.6% and 1.8%.^5^, ^6^, ^7^, ^8^ Patients having abdominal surgery are also particularly vulnerable to developing postoperative pneumonia, with an estimated incidence of 3.2% to 10.7%.^9^, ^10^, ^11^, ^12^, ^13^, ^14^, ^15^ Postoperative pneumonia is associated with increased mortality, length of hospital stay and cost of care. Thompson et al^9^ reported that postoperative pneumonia increased the risk of in‐hospital mortality ninefold, resulting in a mean increase of 11 days in length of hospital stay and a $28 000 US dollar increase in total hospital costs per patient. A better understanding of which patients are at an increased risk of postoperative pneumonia is important to help prioritize the introduction of enhanced preventive interventions.^6^

In a study of pulmonary complications specific to pneumonia, Kinlin et al^2^ reported a clinical prediction rule for use after coronary artery bypass graft surgery. Gupta et al^5^ developed a risk calculator in patients who underwent multiple surgical subspecialties (cardiac surgery 0.3%). Seven independent predictors were identified: age; American Society of Anesthesiologists (ASA) class; chronic obstructive pulmonary disease; dependent functional status; preoperative sepsis; smoking; and type of operation. Arozullah et al^8^ also developed a multifactorial risk index for predicting postoperative pneumonia after major non‐cardiac surgery. Pneumonia rates were 0.2% among those with 0‐15 risk points; 1.2% for those with 16‐25 points; 4.0% for those with 26‐40 points; 9.4% for those with 41‐55 points; and 15.3% for those with >55 points. Several models for wide‐spectrum pulmonary complications, including pulmonary collapse or pulmonary embolism post‐abdominal surgery, have been reported;^10^, ^13^, ^16^ however, a risk calculator to predict pneumonia after abdominal surgery is not currently available.

Enhanced preventive interventions are required in patients who are assessed as high risk for postoperative pneumonia. Pneumonia‐prevention programs have been successfully implemented not only in intensive care unit (ICU) settings but also in surgical wards.^6^, ^17^ The program in surgical wards consists of ambulation, breathing exercises, oral care, and bedhead elevation.^6^ However, the pneumonia‐prevention program has not been implemented routinely in patients undergoing gastroenterological surgery because of the lack of a distribution of the standard approach in non‐critical care settings and the limited number of medical staff in Japan. In addition, a concerted effort is needed to maintain compliance with the program. To become a widely adopted program, identification of high‐risk patients for postoperative pneumonia is required.

The aim of the present study was to develop and validate a risk calculator for predicting postoperative pneumonia after gastroenterological surgery. We used data from the gastroenterological section of the National Clinical Database (NCD) of Japan, which was established in April 2010, with 10 surgical subspecialty societies on the board of the Japan Surgical Society.^18^ The NCD collaborates with the American College of Surgeons' National Surgical Quality Improvement Program (ACS‐NSQIP),^19^ which shares a similar goal of developing a standardized surgical database for quality improvement.^20^

## 2 METHODS

### 2.1 Data set

The NCD is a nationwide project administered in conjunction with the board certification system for surgery in Japan. Data were extracted from the 2011‐2013 NCD data files. There were 2158 participant hospitals. The NCD continuously recruits individuals who approve the data, and members of various departments are in charge of cases and data entry officers, and a web‐based data management system ensures data traceability. The staff also validate data consistency through random inspections of the institutions. Surgical cases from each department were registered in the gastroenterological surgery section of the NCD, which required detailed input of eight selected main procedures. All variables and definitions, as well as the inclusion criteria for the NCD, are accessible on the NCD web site (http://www.ncd.or.jp/). The NCD supports an E‐learning system to ensure consistent data entry.

### 2.2 Patients

Inclusion criteria for the study were patients who underwent the following operations: (i) esophagectomy; (ii) total gastrectomy, (iii) distal gastrectomy; (iv) right colectomy; (v) low anterior resection; (vi) hepatectomy with >1 segment except for the lateral segment; (vii) pancreaticoduodenectomy; and (viii) surgery for acute diffuse peritonitis. Patients agreed for their data to be included in the research projects by using presumed consent with an opt‐out through the web page and/or a notice from each hospital. The NCD project was approved by the Japan Surgical Society Ethics Committee on November 2010. Patients who declined to have their records entered into the NCD were excluded from our analysis. Records with missing data on patient age, gender, or the occurrence of postoperative pneumonia were excluded. In the 2011‐12 dataset for the development of the risk calculator, 247 604 records were used; in the 2013 dataset for the validation of the model, 134 520 records were used.

### 2.3 Outcome measures

Primary outcome of interest was postoperative pneumonia. Postoperative pneumonia was defined as pneumonia occurring within 30 days post‐surgery in patients with no evidence of pneumonia preoperatively.

The registry defines postoperative pneumonia as having met one of the following conditions:

1 Rales or dullness to percussion on physical examination of the chest and any of the following:
  - New onset of purulent sputum or change in character of sputum.
  - Positive growth in blood culture.
  - Isolation of pathogen from specimen obtained by transtracheal aspirate, bronchial brushing, or biopsy.
2 Chest radiography showing new or progressive infiltrate, consolidation or pleural effusion, and any of the following:
  - New onset of purulent sputum or change in character of sputum.
  - Positive growth in blood culture.
  - Isolation of pathogen from specimen obtained by transtracheal aspirate, bronchial brushing, or biopsy.
  - Isolation of virus or detection of viral antigen in respiratory secretions.
  - Diagnostic single antibody titer (IgM) or fourfold increase in paired serum samples (IgG) for pathogen.
  - Histopathological evidence of pneumonia.

In addition, we evaluated 30‐day mortality and operative mortality in patients with pneumonia. The latter was defined within the index hospitalization period, regardless of the length of hospital stay (up to 90 days), as well as any death after discharge, within 30 days of surgery. Surgical site‐related morbidities that occurred within 30 days of surgery (superficial/deep incisional SSI, organ space SSI, wound dehiscence, and anastomotic leak) were also assessed.

### 2.4 Preoperative and intraoperative variables

NCD variables including patient demographics, pre‐existing comorbidities, preoperative laboratory values and perioperative data are almost identical to those applied in ACS‐NSQIP.^21^ In addition to these, intraoperative factors such as prolonged surgery and severe blood loss were also evaluated. The definition of prolonged surgery is an operation lasting over the 75th percentile of the distribution of operation time for a specific category of procedures. The definition of severe blood loss is blood loss that is over the 75th percentile of the distribution for a specific category of procedures.

### 2.5 Statistical analysis

IBM SPSS Statistics for Windows (Version 20; IBM Corp, Armonk, NY, USA) was used for all data analysis. Univariate analysis of the data was carried out using Fisher's exact test, unpaired Student's *t* test, and Mann‐Whitney *U* test. Data were assigned to one of two sets: model development was based on the 2011‐12 dataset; and the model was validated using the 2013 dataset.

Logistic regression models were constructed using stepwise selection of the predictors. Discriminatory ability of the prediction rule in the derivation group was quantified using the area under the receiver‐operating characteristic (ROC) curve. Model calibration was examined by comparing the observed and predicted means with 10 equally sized subgroups, which were arranged in order of increasing patient risk. Model validation applied the developed model to estimate pneumonia probabilities for all patients in the 2013 dataset. A value of *P* < 0.05 was considered statistically significant.

## 3 RESULTS

Among 382 124 patients in the 2011‐13 dataset, postoperative pneumonia was observed in 11 105 patients (2.9%). Patients who experienced pneumonia had a significantly higher operative mortality (23.1% vs 2.1%, *P* < 0.001). Operative mortality in patients with pneumonia was 12.5% in esophagectomy, 20.2% in total gastrectomy, 16.6% in distal gastrectomy, 28.4% in right colectomy, 17.8% in low anterior resection, 35.6% in hepatectomy, 27.6% in pancreaticoduodenectomy, and 43.0% in surgery for acute diffuse peritonitis.

Patients with postoperative pneumonia had more surgical site‐related complications compared with those without postoperative pneumonia. Specifically, 23.1% of patients with postoperative pneumonia also had a superficial incisional SSI; 25.1% had an organ/space SSI; and 20.4% had an anastomotic leak. The most common types of surgery were gastrectomy [total gastrectomy: n = 58 809 (15.4%); and distal gastrectomy: n = 112 867 (29.5%)] and colorectal resection [right colectomy: n = 60 738 (15.9%); and lower anterior resection: n = 58 401 (15.3%)] followed by pancreaticoduodenectomy [n = 27 702 (7.2%)], operation for acute diffuse peritonitis [n = 27 377 (7.2%)], hepatectomy [n = 23 610 (6.2%)] and esophagectomy [n = 16 556, (4.3%)]. Cut‐off values for prolonged surgery and severe intraoperative blood loss are shown in Table 1.

**Table 1:** Cut‐off values of prolonged surgery and severe intraoperative blood loss in eight selected gastroenterological surgeries

Surgical procedure	Duration of surgery, 75th percentile (min)	Intraoperative blood loss, 75th percentile (mL)
Esophagectomy	573	684
Distal gastrectomy	305	323
Total gastrectomy	338	635
Right hemicolectomy	245	246
Low anterior resection	335	375
Hepatectomy with >1 segment except for the lateral segment	477	1570
Pancreaticoduodenectomy	547	1300
Operation for acute diffuse peritonitis	160	247

The highest incidence of postoperative pneumonia was observed in esophagectomy (13.7%). Surgery for acute diffuse peritonitis was next highest, with an incidence of 7.5%. Gastrectomy (total/distal), pancreaticoduodenectomy and hepatectomy had incidences of 2.5% (3.6/1.9), 2.5% and 2.2%, respectively. The lowest incidence of postoperative pneumonia was observed in low anterior resection (0.9%) and right hemicolectomy (1.6%).

**Figure 1:**
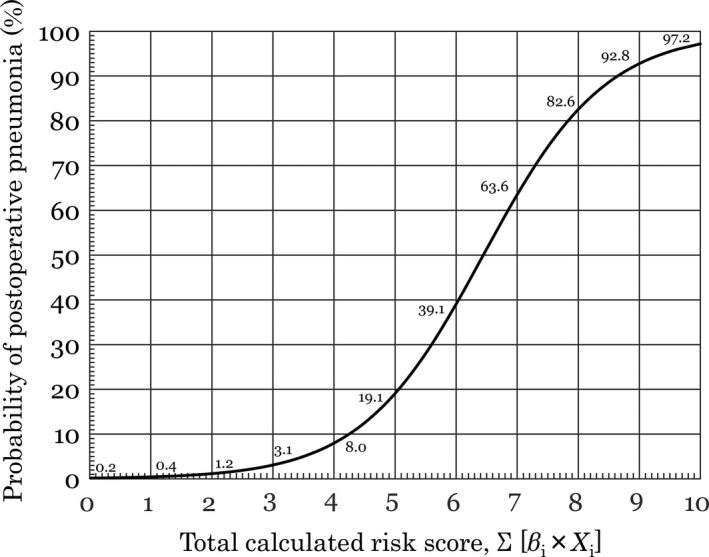
Approximated predicted risk for postoperative pneumonia. Risk for postoperative pneumonia is approximated from a nomogram of predicted probability vs calculated total risk score. β_i_ is the coefficient of the variable X_i_ in the logistic regression equation provided in Table 4

Univariate analysis for predictors of postoperative pneumonia is shown in Table 2. We identified 38 independent predictors of postoperative pneumonia, and the multivariate logistic regression model with odds ratios (OR), 95% confidence intervals (CI) and β coefficient is shown in Table 3. After the input of significant primary disease and surgical procedures for a given patient (shown in Table 4), characteristics of 18 patients including gender, chronic obstructive pulmonary disease, sepsis and need for any assistance in the activities of daily living (ADL), six preoperative laboratory data and two intraoperative factors were used for risk calculation (Table 5). The scoring system for the postoperative pneumonia risk models based on the logistic regression equation was as follows:

$$\text{Probability of pneumonia}=\frac{A}{1+A}$$

$$A=e(-6.444+\sum \left[\beta_{i}X_{i}\right]),$$

where β_*i*_ is the coefficient of the variable *X*_*i*_ in the logistic regression equation provided in Table 4. If a categorical risk factor is present, the *X*_*i*_ value is 1 (0 if it is absent). For the age categories, *X*_*i*_ is defined according to the following definitions (<60 years = 1, 60‐64 years = 2, 65‐69 years = 3, 70‐74 years = 4, 75‐79 years = 5, ≥80 years = 6). Total risk score for a given patient was calculated by summing (β_*i*_ × *X*_*i*_) of the primary diagnosis and surgical procedures (Table 4) and patients' characteristics, laboratory data and intraoperative factors (Table 5). (−6.444 + Σ [β_*i*_ × *X*_*i*_]) represents the log of the odds of pneumonia and −6.444 is the intercept of the multivariate model. Risk of postoperative pneumonia can also be approximated from a graph of predicted probability versus calculated risk score (Σ [β_*i*_ × *X*_*i*_]) (Figure 1).

**Table 2:** Univariate analysis of risk factors for postoperative pneumonia in 382 124 patients undergoing eight main gastroenterological surgeries between 2011 and 2013 using the NCD in Japan

Risk factors	No. of patients with postoperative pneumonia (%)		
	Corresponding factor (+)	Corresponding factor (−)	*P* value
Demographics			
Age category (<60, 60‐64, 65‐69, 70‐74, 75‐79, ≥80 y)	–		<0.0001
Male	8864/248 341 (3.6%)	2241/133 783 (1.7%)	<0.0001
Comorbidity			
Chronic obstructive pulmonary disease	1245/13 166 (9.5%)	9860/368 958 (2.7%)	<0.0001
Cerebrovascular disease	1048/13 979 (7.5%)	10 057/368 145 (2.7%)	<0.0001
Diabetes	2352/6671 (3.5%)	8753/315 413 (2.8%)	<0.0001
Ascites	1018/12 871 (7.9%)	10 087/369 253 (2.7%)	<0.0001
Hypertension	4896/132 237 (3.7%)	6209/249 887 (2.5%)	<0.0001
Congestive heart failure	320/3369 (9.5%)	10 785/378 755 (2.8%)	<0.0001
Myocardial infarction	125/2101 (5.9%)	10 980/380 023 (2.9%)	<0.0001
Angina	246/5095 (4.8%)	10 859/377 029 (2.9%)	<0.0001
Peripheral vascular disease	136/1365 (10.0%)	10 969/380 759 (2.9%)	<0.0001
Hemodialysis	239/3564 (6.7%)	10 866/378 560 (2.9%)	<0.0001
Bleeding disorder	1129/15 090 (7.5%)	9976/367 034 (2.7%)	<0.0001
ASA Physical Status classification >grade 2	3549/50 276 (7.1%)	7566/331 848 (2.3%)	<0.0001
Medical history			
Previous cardiac surgery	266/4354 (6.1%)	10 839/377 770 (2.9%)	<0.0001
Previous percutaneous coronary intervention	532/8906 (6.0%)	10 573/373 218 (2.8%)	<0.0001
Chronic steroid use	309/3950 (7.8%)	10 796/378 174 (2.9%)	<0.0001
Preoperative chemotherapy	692/11 627 (6.0%)	10 413/370 497 (2.8%)	<0.0001
Preoperative radiotherapy	204/3381 (6.0%)	10 901/378 743 (2.9%)	<0.0001
Lifestyle, activity			
ADL need for any assistance	2505/25 384 (9.7%)	8600/356 290 (2.4%)	<0.0001
Tobacco use, Brinkman index >400	4635/109 161 (4.2%)	6470/272 963 (2.4%)	<0.0001
Alcohol habitually	3525/97 146 (3.6%)	7580/284 978 (2.7%)	<0.0001
Preoperative particular condition			
Body weight loss >10%	1182/20 154 (5.9%)	9923/361 970 (2.7%)	<0.0001
Preoperative transfusions	676/10 387 (6.5%)	10 429/371 737 (2.8%)	<0.0001
Disseminated cancer	534/11 954 (4.5%)	10 571/370 170 (2.9%)	<0.0001
SIRS with infection	1322/9798 (13.5%)	9783/372 326 (2.6%)	<0.0001
Emergent surgery	2410/35 199 (6.8%)	8695/346 925 (2.5%)	<0.0001
Preoperative ventilation	214/1491 (14.4%)	10 891/380 633 (2.9%)	<0.0001
Primary disease			
Gastric cancer	4233/164 858 (2.6%)	6872/217 266 (3.2%)	<0.001
Cholangiocarcinoma (extrahepatic bile ducts perihilar)	75/1838 (4.1%)	11 030/380 286 (2.9%)	0.0031
Cholangiocarcinoma (extrahepatic bile ducts distal)	204/5817 (3.5%)	10 901/376 307 (2.9%)	0.0067
Gallbladder cancer	49/1198 (4.1%)	11 056/380 926 (2.9%)	0.0163
No tumor	2073/34 530 (6.0%)	9032/347 594 (2.6%)	<0.001
Primary surgery			
Operation for acute diffuse peritonitis (JSGS15)	2045/27 377 (7.5%)	9060/354 747 (2.6%)	<0.001
Hepatectomy with >1 segment except for the lateral segment (JSGS39)	515/23 610 (2.2%)	10 590/358 514 (3.0%)	<0.001
Pancreaticoduodenectomy (JSGS91)	692/27 702 (2.5%)	10 413/354 422 (2.9%)	<0.001
Esophagectomy (JSGS103)	2268/16 556 (13.7%)	8837/365 568 (2.4%)	<0.001
Low anterior resection (JSGS 3)	524/58 401 (0.9%)	10 581/323 723 (3.3%)	<0.001
Right hemicolectomy (JSGS 31)	1000/60 738 (1.6%)	10 105/321 386 (3.1%)	<0.001
Total gastrectomy (JSGS47)	2105/58 809 (3.6%)	9000/323 315 (2.8%)	<0.001
Distal gastrectomy (JSGS49)	2192/112 867 (1.9%)	8913/269 257 (3.3%)	<0.001
Associated surgery			
Splenectomy (JSGS69)	225/5873 (3.8%)	10 880/376 251 (2.9%)	<0.001
Enterostomy/closure with bowel resection (JSGS73)	353/5640 (6.3%)	10 752/376 484 (2.9%)	<0.001
Enterostomy/closure without bowel resection (JSGS74)	685/10 066 (6.8%)	10 420/372 058 (2.8%)	<0.001
Preoperative laboratory examination			
Albumin <2.5 g/dL	1283/13 542 (9.5%)	9822/368 582 (2.7%)	<0.001
AST >35 IU/L	2100/48 875 (4.3%)	9005/333 249 (2.7%)	<0.001
ALP >340 IU/L	1877/48 829 (3.8%)	9228/333 295 (2.8%)	<0.001
BUN >25 mg/dL	2103/27 217 (7.7%)	9002/354 907 (2.5%)	<0.001
Na <138 mEq/dL	2636/48 885 (5.4%)	8469/333 239 (2.5%)	<0.001
Creatinine >3 mg/dL	396/5575 (7.1%)	10 709/376 549 (2.8%)	<0.001
Hematocrit <21%	150/2886 (5.2%)	10 955/379 238 (2.9%)	<0.001
Hematocrit: male >48%, female >42%	234/101 866 (2.3%)	10 871/371 938 (2.9%)	0.002
Total bilirubin >3 mg/dL	275/6210 (4.4%)	10 830/375 914 (2.9%)	<0.001
C‐reactive protein >10 mg/dL	1401/17 665 (7.9%)	9704/364 459 (2.7%)	<0.001
White blood cell count <3500/μL	1025/23 487 (4.4%)	10 090/358 637 (2.8%)	<0.001
White blood cell count >12 000/μL	945/16 817 (5.6%)	10 160/365 307 (2.8%)	<0.001
Platelet count <15 × 10^4^/μL	1695/32 917 (5.1%)	9410/349 207 (2.7%)	<0.001
Prothrombin time‐INR >1.1	2742/51 603 (5.3%)	8363/330 521 (2.5%)	<0.001
Intraoperative factors			
Prolonged surgery (>75th percentile)	3706/95 328 (3.9%)	7399/286 796 (2.6%)	<0.001
Severe blood loss (>75th percentile)	4319/94 332 (4.6%)	6786/287 792 (2.4%)	<0.001

ADL, activities of daily living; ALP, alkaline phosphatase; ASA, American Society of Anesthesiologists; AST, aspartate aminotransferase; BUN, blood urea nitrogen; INR, international normalized ratio; NCD, National Clinical Database; SIRS, systemic inflammatory response syndrome.

**Table 3:** Multivariate logistic regression model of risk factors for postoperative pneumonia in 247 604 patients between 2011 and 2012 using the NCD in Japan

Risk factors	Multivariate analysis				
	Adjusted odds ratio	95% CI		*P* value	β coefficient
		Lower	Upper		
Demographics					
Age category	1.293	1.272	1.314	<0.001	0.257
Male	1.740	1.632	1.856	<0.001	0.554
Comorbidity					
Chronic obstructive pulmonary disease	2.041	1.877	2.219	<0.001	0.713
Cerebrovascular disease	1.607	1.469	1.758	<0.001	0.474
Diabetes	1.073	1.009	1.140	0.024	0.070
Ascites	1.123	1.014	1.244	0.026	0.116
Hypertension	1.114	1.059	1.172	<0.001	0.108
Bleeding disorder	1.129	1.030	1.237	0.010	0.121
ASA >grade 2	1.417	1.329	1.511	<0.001	0.348
Medical history					
Previous percutaneous coronary intervention	1.301	1.154	1.467	<0.001	0.263
Chronic steroid use	1.626	1.391	1.901	<0.001	0.486
Lifestyle, activity					
ADL need for any assistance	2.066	1.921	2.222	<0.001	0.726
Tobacco use, Brinkman index >400	1.305	1.236	1.379	<0.001	0.266
Preoperative particular condition					
Body weight loss >10%	1.286	1.183	1.397	<0.001	0.251
Preoperative transfusions	1.204	1.079	1.343	0.001	0.185
SIRS with infection	1.777	1.594	1.981	<0.001	0.575
Emergent surgery	1.609	1.423	1.818	<0.001	0.476
Disseminated cancer	1.187	1.056	1.333	0.004	0.171
Primary disease					
Gastric cancer (total/distal gastrectomy, JSGS47, 49)	1.843	1.722	1.973	<0.001	0.612
Cancer of small intestine	1.606	1.163	2.218	0.004	0.474
Cholangiocarcinoma (extrahepatic bile ducts perihilar)	1.655	1.255	2.184	<0.001	0.504
Cholangiocarcinoma (extrahepatic bile ducts distal)	1.284	1.043	1.580	0.018	0.250
Gallbladder cancer	1.655	1.165	2.351	0.005	0.504
Primary surgery					
Operation for acute diffuse peritonitis (JSGS15)	1.740	1.524	1.986	<0.001	0.554
Hepatectomy with >1 segment except for lateral segment (JSGS39)	1.700	1.499	1.927	<0.001	0.530
Pancreaticoduodenectomy (JSGS91)	1.809	1.590	2.057	<0.001	0.593
Esophagectomy (JSGS103)	13.727	12.640	14.907	<0.001	2.619
Associated surgery					
Splenectomy (JSGS69)	1.363	1.138	1.632	0.001	0.310
Enterostomy/closure with bowel resection (JSGS73)	1.279	1.094	1.495	0.002	0.246
Enterostomy/closure without bowel resection (JSGS74)	1.228	1.097	1.375	<0.001	0.206
Preoperative laboratory examination					
Hematocrit: male >48%, female >42%	1.178	1.006	1.379	0.042	0.164
Albumin <2.5 g/dL	1.365	1.244	1.498	<0.001	0.311
AST >35 IU/L	1.130	1.056	1.210	<0.001	0.123
ALP >340 IU/L	1.146	1.066	1.232	<0.001	0.136
BUN >25 mg/dL	1.232	1.145	1.325	<0.001	0.208
Na <138 mEq/dL	1.168	1.098	1.241	<0.001	0.155
Intraoperative factors					
Prolonged surgery (>75th percentile)	1.368	1.295	1.444	<0.001	0.313
Severe blood loss (>75th percentile)	1.461	1.387	1.539	<0.001	0.379

95% CI, 95% confidence interval; ADL, activities of daily living; ALP, alkaline phosphatase; ASA, American Society of Anesthesiologists; AST, aspartate aminotransferase; BUN, blood urea nitrogen; NCD, National Clinical Database; SIRS, systemic inflammatory response syndrome.

**Table 4:** Calculation of risk score of primary diagnosis and surgical procedures

Risk factors	*X*_*i*_		β_*i*_
	Yes	No	
1. Disseminated cancer	1	0	0.171
2. Gastric cancer	1	0	0.612
3. Cancer of small intestine	1	0	0.474
4. Cholangiocarcinoma (extrahepatic bile ducts perihilar)	1	0	0.504
5. Cholangiocarcinoma (extrahepatic bile ducts distal)	1	0	0.250
6. Gallbladder cancer	1	0	0.504
7. Operation for acute diffuse peritonitis	1	0	0.554
8. Hepatectomy with >1 segment except for lateral segment	1	0	0.530
9. Pancreaticoduodenectomy	1	0	0.593
10. Esophagectomy	1	0	2.619
11. Splenectomy	1	0	0.310
12. Enterostomy/closure with bowel resection	1	0	0.246
13. Enterostomy/closure without bowel resection	1	0	0.206

Risk score of primary diagnosis and surgical procedures, Σ [β_*i*_ × *X*_*i*_].

β_*i*_ is the coefficient of the variable *X*_*i*_ in the logistic regression equation. If a categorical risk factor is present, the *X*_*i*_ value is 1 (0 if it is absent). A total risk score of primary diagnosis and surgical procedures for a given patient is calculated by summing β_*i*_ × *X*_*i*_.

**Table 5:** Calculation of the total risk score

Risk factors	*X*_*i*_		β_*i*_
	Yes	No	
Patient characteristics			
1. Age category, y	<60 = 1, 60‐64 = 2, 65‐69 = 3, 70‐74 = 4, 75‐79 = 5, ≥80 = 6		0.257
2. Male	1	0	0.554
3. Chronic obstructive pulmonary disease	1	0	0.713
4. Cerebrovascular disease	1	0	0.474
5. Diabetes	1	0	0.070
6. Ascites	1	0	0.116
7. Hypertension	1	0	0.108
8. Bleeding disorder	1	0	0.121
9. ASA >grade 2	1	0	0.348
10. Previous percutaneous coronary intervention	1	0	0.263
11. Chronic steroid use	1	0	0.486
12. ADL need for any assistance	1	0	0.726
13. Tobacco use, Brinkman index >400	1	0	0.266
14. Body weight loss >10%	1	0	0.251
15. Preoperative transfusions	1	0	0.185
16. SIRS with infection	1	0	0.575
17. Emergent surgery	1	0	0.476
Preoperative laboratory data			
1. Hematocrit: male >48%, female >42%	1	0	0.164
2. Albumin <2.5 g/dL	1	0	0.311
3. AST >35 IU/L	1	0	0.123
4. ALP >340 IU/L	1	0	0.136
5. BUN >25 mg/dL	1	0	0.208
6. Na <138 mEq/dL	1	0	0.155
Intraoperative factors			
1. Prolonged surgery (>75th percentile)	1	0	0.313
2. Severe blood loss (>75th percentile)	1	0	0.379

Risk score of patients' characteristics, laboratory data and intraoperative factors, Σ [β_*i*_ × *X*_*i*_].

Total risk score = risk score of primary diagnosis and surgical procedures + risk score of patients' characteristics, laboratory data and intraoperative factors.

β_*i*_ is the coefficient of the variable *X*_*i*_ in the logistic regression equation. If a categorical risk factor is present, the *X*_*i*_ value is 1 (0 if it is absent). For the age categories, *X*_*i*_ is defined according to the definition. Total risk score for a given patient is calculated by summing β_*i*_ × *X*_*i*_.

ADL, activities of daily living; ALP, alkaline phosphatase; ASA, American Society of Anesthesiologists; AST, aspartate aminotransferase; BUN, blood urea nitrogen; SIRS, systemic inflammatory response syndrome.

**Figure 2:**
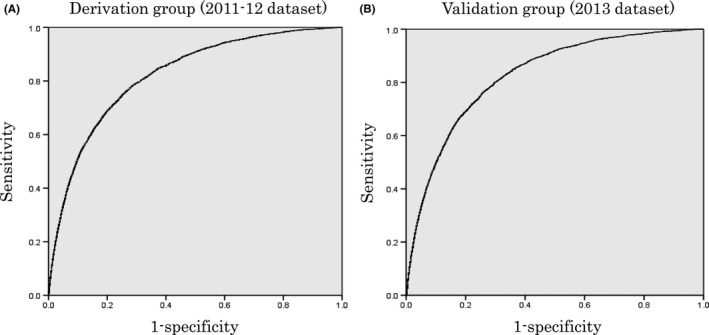
Receiver‐operating characteristic (ROC) curve for postoperative pneumonia in the derivation and validation groups. Area under the ROC curve was 0.822 (95% CI, 0.817‐0.826) in the derivation subset of the study sample and 0.826 (95% CI, 0.819‐0.832) in the validation subset of the study sample

**Figure 3:**
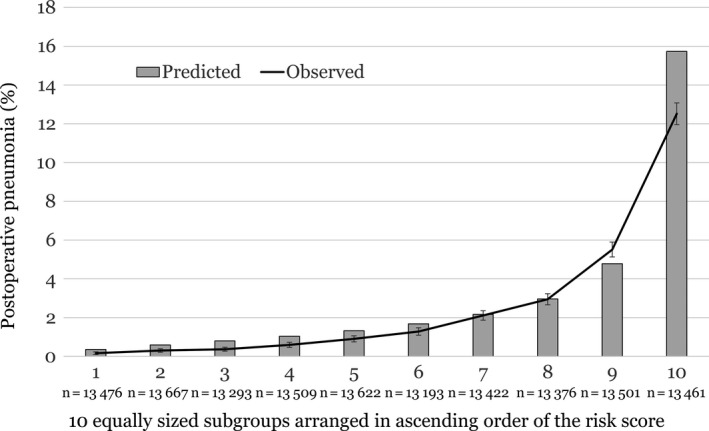
Calibration of the postoperative pneumonia model. Bar charts represent mean rate for the predicted events. Line chart represents those for the observed events, and the error bars represent 95% CI of the observed events. The risk score is calculated using Σ [β_*i*_ × *X*_*i*_]. β_i_ is the coefficient of the variable X_i_ in the logistic regression equation. This figure represents how well the rates of the predicted events matched those of the observed events according to the patient risk subgroups

Area under the ROC curve was 0.822 (95% CI, 0.817‐0.826; *P* < 0.001) in the derivation group, indicating good discrimination ability (Figure 2). In the analysis of the 2013 dataset for the validation of the model (n = 134 520), the area under the ROC curve was 0.826 (95% CI, 0.819‐0.832) (Figure 2). Figure 3 shows the calibration of the models. The rate for the predicted events corresponded reasonably well with those for the observed events in each risk‐stratified subgroup.

## 4 DISCUSSION

Estimations of the incidence of postoperative pneumonia vary widely in the literature, from 0.6% to 17.5%.^2^, ^3^, ^4^, ^5^, ^6^, ^7^, ^8^, ^9^, ^10^, ^11^, ^12^, ^13^, ^14^, ^15^, ^22^ The variability is primarily because of the diagnosis criteria used and differing surgical procedures. In the present study, the overall incidence of postoperative pneumonia within 30 days of gastroenterological surgery was 2.9%, which is similar to the incidence reported by Yang et al^10^ (3.2%) in a sample of 165 196 patients who underwent major abdominal surgery. Thompson et al^9^ reported that hospital‐acquired pneumonia occurred in 10.7% of patients following intra‐abdominal surgery. They analyzed all patients with a discharge diagnosis of pneumonia, including late‐onset pneumonia.

In the present study, the highest incidence of postoperative pneumonia was observed after esophagectomy (13.7%) and the lowest incidence was observed after low anterior resection and right hemicolectomy (0.9% and 1.6%, respectively). Yang et al^10^ described similar incidences as follows; esophagectomy 16.2%; gastrectomy 6.4%; pancreatectomy 4.8%; hepatectomy 3.3%; and colectomy/proctectomy 2.4%. In Japan, transthoracic excision of the esophagus with extended lymph node dissection is the standard procedure for patients with esophageal cancer. In one report from Japan, the incidence of pneumonia was 8.7% in patients who underwent subtotal esophagectomy.^14^ Molena et al^15^ reported a relatively low incidence of postoperative pneumonia (5.9%), possibly because a considerable number of patients underwent transhiatal esophagectomy. In a study of pulmonary complications, those who had esophagectomy remained prevalent despite advances in perioperative management.^14^, ^15^ In addition, upper abdominal surgical location has been consistently identified as an independent risk factor for postoperative pulmonary complications.^13^, ^16^, ^23^

In the multivariate logistic regression analysis, a high β coefficient was observed in males, patients with chronic obstructive pulmonary disease (COPD), patients with a need for any assistance in ADL, patients with preoperative systemic inflammatory response syndrome with infection, three diseases including gastric cancer, gallbladder cancer and cholangiocarcinoma (extrahepatic bile ducts perihilar) and four operative procedures including esophagectomy, pancreaticoduodenectomy, operation for acute diffuse peritonitis and hepatectomy with >1 segment except for the lateral segment. These were consistent with previously described risk factors for postoperative pneumonia. Gupta et al^5^ reported that preoperative variables associated with an increased risk of postoperative pneumonia included age, ASA class, COPD, dependent functional status, preoperative sepsis, smoking and type of operation; all these risk factors were also included in our risk model. Arozullah et al^8^ also developed a postoperative pneumonia risk index that included type of surgery, age, functional status, weight loss, COPD, general anesthesia, impaired sensorium, cerebral vascular accident, blood urea nitrogen level, transfusion, emergency surgery, steroid use, smoking and alcohol use. Using 38 variables, our risk calculator indicated good discrimination ability and was well calibrated. The model also showed reasonable discrimination in the validation subset.

As a practical use of our risk model, tentative probability of postoperative pneumonia is estimated by the preoperative risk score, calculated by summing the β_*i*_ × *X*_*i*_ of primary diagnosis and surgical procedures, patient characteristics and preoperative laboratory data. Using these data, an attending physician explains the risk of postoperative pneumonia to patients and the indication for a pneumonia‐prevention program is discussed among medical staff. After surgery, definitive probability of postoperative pneumonia is assessed by adding the risk score of intraoperative factors to the preoperative risk score.

Our risk model for postoperative pneumonia addresses several limitations of previous studies. Previous studies have had relatively small sample sizes. The Japanese NCD contains data from 2158 participant hospitals, including both community and teaching hospitals. We developed the risk model limited to patients undergoing gastroenterological surgery. Previous studies included patients undergoing a wide range of non‐cardiac operations.^5^, ^7^, ^8^ Most of the previous studies have included a wide array of postoperative pulmonary complications such as atelectasis, pulmonary embolism and respiratory failure.^10^, ^13^, ^16^, ^22^ Varying definitions of postoperative pulmonary complications cause variability in risk estimates. We evaluated preoperative risk factors as well as intraoperative factors (prolonged surgery and severe hemorrhage). Several studies have identified that these two intraoperative factors were independently related to postoperative pneumonia^7^, ^21^ or respiratory complication.^15^, ^24^

Despite the many strengths of our study, there are several limitations. First, although our model was validated by the 2013 dataset, external validation on independent datasets from other countries is required before the index can be widely applied in clinical practice. Second, because a substantial number of cancer patients were registered in gastroenterological surgery, influence of cancer stage and extent of lymphadenectomy should also be considered. Third, pulmonary function test results were not available in this study. Fourth, laparoscopic approach could reduce pulmonary complications in several procedures.^25^, ^26^, ^27^ However, we did not evaluate laparoscopic surgery as a potential predictor. Fifth, postoperative pneumonia was defined as pneumonia occurring within 30 days post‐surgery. Pneumonia rate will be underestimated if no post‐discharge surveillance is carried out. Adherence of post‐discharge surveillance might be different in each institution. Finally, information on hospital volume is not contained in the database.

## 5 CONCLUSIONS

We developed a risk calculator for predicting postoperative pneumonia after gastroenterological surgery using a large dataset from the NCD in Japan. Performance of the model was good in terms of discrimination and calibration, and the model was validated using datasets submitted to the NCD in different years. In patients who were assessed as high risk for postoperative pneumonia, enhanced preventive interventions should be considered. This risk model is also useful in counseling and for obtaining informed consent from patients.

## DISCLOSURE

Conflicts of Interest: Authors declare no conflicts of interest for this article.
